# Anti-Inflammatory Effects of Encapsulated Human Mesenchymal Stromal/Stem Cells and a Method to Scale-Up Cell Encapsulation

**DOI:** 10.3390/biom12121803

**Published:** 2022-12-02

**Authors:** Suneel Kumar, Maciej Kabat, Sayantani Basak, Joanne Babiarz, Francois Berthiaume, Martin Grumet

**Affiliations:** 1 Department of Biomedical Engineering, Rutgers, The State University of New Jersey, Piscataway, NJ 08854, USA; 2 W. M. Keck Center for Collaborative Neuroscience, Rutgers Stem Cell Research Center, Department of Cell Biology and Neuroscience, Rutgers, The State University of New Jersey, Piscataway, NJ 08854, USA

**Keywords:** mesenchymal stem cells, spinal cord injury, encapsulation, alginate, human IL10

## Abstract

Mesenchymal stem/stromal cells (MSC) promote recovery in a wide range of animal models of injury and disease. They can act in vivo by differentiating and integrating into tissues, secreting factors that promote cell growth and control inflammation, and interacting directly with host effector cells. We focus here on MSC secreted factors by encapsulating the cells in alginate microspheres, which restrict cells from migrating out while allowing diffusion of factors including cytokines across the capsules. One week after intrathecal lumbar injection of human bone marrow MSC encapsulated in alginate (eMSC), rat IL-10 expression was upregulated in distant rat spinal cord injury sites. Detection of human IL-10 protein in rostrally derived cerebrospinal fluid (CSF) indicated distribution of this human MSC-secreted cytokine throughout rat spinal cord CSF. Intraperitoneal (IP) injection of eMSC in a rat model for endotoxemia reduced serum levels of inflammatory cytokines within 5 h. Detection of human IL-6 in sera after injection of human eMSC indicates rapid systemic distribution of this human MSC-secreted cytokine. Despite proof of concept for eMSC in various disorders using animal models, translation of encapsulation technology has not been feasible primarily because methods for scale-up are not available. To scale-up production of eMSC, we developed a rapid, semi-continuous, capsule collection system coupled to an electrosprayer. This system can produce doses of encapsulated cells sufficient for use in clinical translation.

## 1. Introduction

Mesenchymal stem/stromal cells (MSC) have gained great interest as new medical treatments. Clinical development of MSC therapies is based on extensive studies in ani-mal models for human disorders and diseases demonstrating improved outcomes [1]. MSC can act by three major classes of mechanisms [1] differentiation into different types of cell lineages and integration into tissues, which have applications for regenerative medicine, [2] MSC direct contact with host cells to modulate functions of effector cells, and [3] secretion of factors including those that promote cell survival and growth, and cytokines that modulate inflammation and immune cell function [2,3]. Proof of concept for efficacy of MSC in the clinic has been demonstrated for Graft vs. Host Disease, which is believed to involve one or both latter two mechanisms by modulating cytokine storm and inhibiting inflammation [4,5]. However, the functional roles and fates of MSC differentiation and integration after injection into humans have not been elucidated. Understanding mechanisms of MSC action, which has been difficult in vivo even in animal models, will facilitate improved treatments for translational studies [2,3].

Cytokine storm occurs in injuries and diseases that have persistent highly elevated levels of pro-inflammatory cytokines [6]. Cytokine storm often occurs in sepsis with inflammatory responses to pathogens [7], which can lead to multiple organ failure with a mortality rate of >25% [8]. The human survival rate has decreased to this level over the past several decades primarily due to improved diagnosis and more aggressive critical care, however, no new therapies have been developed over decades [9]. MSC are effective in treating sepsis in rodent models [10] by releasing anti-inflammatory cytokines including IL-1ra, IL-4, and IL-10, and prostaglandins, e.g., PGE2, which suppress inflammation and resolve cytokine storms [11].

Cell encapsulation in alginate was initially developed to treat diabetes using islet cells to release insulin [12]. Analysis of encapsulated MSC (eMSC) in vitro has proven that secretion of cytokines and other factors can suppress secretion of pro-inflammatory cytokines from activated immune cells such as macrophages [11]. Although encapsulated islets are functional in vivo for short periods [13], recent modifications including the use of less adhesive alginates, which minimize foreign body reactions, yielded encapsulated islets that release insulin for as long as 9 months in non-human primates [14], providing preclinical feasibility for translation [15].

eMSC promote functional recovery after myocardial infarction [16], hindlimb ischemia [17], and spinal cord injury (SCI) [18,19] in animals. In addition, encapsulated genetically engineered cells secrete bioactive proteins in vivo [20,21,22,23]. Encapsulation in alginate prevents migration of cells out from the capsules, thereby allowing effects of secreted factors to be analyzed without complications due to direct interactions of the encapsulated cells with host cells. In contrast to IV-injected MSC that disappear rapidly [24], encapsulation prolongs MSC survival in vivo for weeks to months [14].

We showed previously that intrathecal injection of eMSC into the cauda equina one day after rat SCI mitigated inflammation and improved functional recovery in SCI [18]. By comparison to empty capsule controls, eMSC increased expression of CD206, a marker for anti-inflammatory M2 macrophages, at ~2 cm from the SCI site at thoracic segments 9–10 [18] and decreased expression of the pro-inflammatory isolectin IB4 expressed on activated microglia and macrophages one week after injection [19].

We report here that localized injection of encapsulated human MSC in a rodent model modulates host cytokine expression within the CNS in SCI. Encapsulation enables localized delivery of MSC, and sustained survival of MSC and secretion in vivo. Given these advantages of eMSC, we have developed a scalable semi-continuous system to generate encapsulated cells in quantities sufficient for clinical translation.

## 2. Materials and Methods

### 2.1. Encapsulated Human MSC (eMSC)

Human bone marrow-derived MSC were purchased (Texas A & M, College Station, TX, USA) and expanded in culture up to ~70% confluence in α-MEM complete media as described previously [18]. MSC were used after passage 4–5. For encapsulation in UP LVG alginate (minimum of 60% α-L-Guluronate, Endotoxins ≤100 Eu/g, PRONOVA, Muiden, The Netherlands), MSC were detached using trypsin-EDTA (Gibco, Waltham, MA, USA) and prepared in suspension in a final concentration of 2.25% (*w*/*v*) alginate (Sigma Aldrich, St. Louis, MO, USA) at a cell density of 4 × 10^6^ cells/mL. A Nisco electrostatic encapsulator was used to prepare capsules by cross-linking in 100 mM CaCl_2_ for experiments with animals. The capsules were washed with PBS and then incubated in 0.05% (*w*/*v*) poly-L-lysine in PBS. Alternatively, an electrosprayer (Spraybase, Cambridge, MA, USA) was used to form capsules by cross-linking in 50–100 mM BaCl_2_ without poly-lysine treatment. The poly-lysine stabilizes the capsules formed in 100 mM CaCl_2_ but is not necessary with the BaCl_2_ treatment because it forms more stable capsules than with CaCl_2_ [25]. The capsules containing cells were re-suspended in cell culture media and incubated upright in 25 cm^2^ tissue culture flasks. Cell viability in the capsules was assessed using a Calcein and Ethidium homodimer assay as described by the manufacturer (ThermoFisher, Waltham, MA, USA); numbers of live cells were counted from projections of confocal microscope images using a LSM 510 as described [19] using at least 10 capsules. Other cells that were encapsulated include GFP + C6 glioma cells grown in DMEM/10% FCS [26] and CHO cells grown in α-MEM/10% FCS [27]. Additional details on encapsulation are found in the Appendix A.

### 2.2. Spinal Cord Injury (SCI) and Capsule Injection

Adult female Sprague Dawley rats (77 ± 2 days old, Taconic, Germantown, NY, USA) were treated under protocols approved by the Animal Care and Use Committee of Rutgers, The State University of New Jersey. For SCI surgery, rats were anesthetized with 2% isoflurane (IsoFlo; Abbott Laboratories, Chicago, IL, USA), the spinal cord was exposed by laminectomy at T9–T10, and then contused by dropping a 10 g rod on the exposed cord from a height of 12.5 mm using a MASCIS Impactor as described [18,28]. One day after SCI, capsules without or with 30,000 human MSC suspended in PBS were injected intrathecally into the cauda equina. Animals were anesthetized after 7 or 42 days, and CSF was collected from the foramen magnum. Rats were euthanized, the spinal cord was exposed by laminectomy, and spinal cord tissue in and around the SCI site was collected and frozen on dry ice. The lumbar spine was exposed by laminectomy and washed with PBS to collect capsules. Capsules were allowed to settle under gravity, washed several times with PBS by resuspension and resettling, and counted in 96 well plates. The eMSC were incubated in α-MEM complete media and assayed for live cells. The diameter of the capsules used for SCI were relatively small (214 ± 27 µm) because small needles were needed to inject into the lumbar region to limit damage.

### 2.3. ELISA and Multiplex Assays

Cultures of 5000 MSC or eMSC were incubated for 16 h ± 1 µg/mL lipopolysaccharide (LPS) and the supernatants were collected for assays [19]. ELISA analyses were performed to measure rat TNF (Biolegend, San Diego, CA, USA), human IL-6 (hIL-6) (Biolegend), human IL-10 (R & D Systems, Minneapolis, MN, USA), and prostaglandin E2 (PGE2) (Cayman Chemical, Ann Arbor, MI, USA) using ELISA kits according to the manufacturer’s instructions. Sera from the LPS-treated rats were analyzed using a bead-based multiplex analysis of 12 cytokines, chemokines, and growth factors according to the manufacturer’s instructions (Bio-Plex Pro™ Rat Cytokine Th1/Th2 Assay #171k1002m). Cytokine data was obtained using a Bio-Plex 200 System (Bio-Rad Laboratories Inc., Hercules, CA, USA). Student *t*-tests were used to determine the SEM.

### 2.4. qRT-PCR

Segments of 5 mm from the SCI site epicenter were homogenized using a Polytron PT-2100 homogenizer for thirty seconds at speed 26 with 10% β-mercaptoethanol in RLT lysate buffer (Qiagen RNeasy Plus Mini Kit). Extracted RNA was analyzed using Applied Biosystem’s High-Capacity cDNA Reverse Transcriptase PCR reaction using the PTC-100 Programmable Thermal Controller by MJ Research, Inc. qRT-PCR reactions were carried out using 200 ng/mL of mRNA with a 7500 Fast Real-Time PCR System by Advanced Biosystems. Primers (GGGGCTTCCTAACTGCTACA; nucleotides 126–107 and CTCCGAGACACTGGAAGGTG nucleotides 41–60) were used to measure levels of IL-10 mRNA (rat gene XM_032915519). We used the relative quantitation method that was configured in the software (delta delta Ct quantitation) with GAPDH as the internal control.

### 2.5. Rat Endotoxemia Model

A rat LPS model for endotoxemia and inflammation was performed as described [29]. Briefly, rats were anesthetized with isoflurane and injected intraperitoneally (IP) with 0.6 mL of 10 mg/kg LPS. After 1 h, capsules in 2 mL of HBSS were IP injected with 18 G needles. Cardiac puncture was performed to collect blood 5 h later under anesthesia and plasma was frozen for assays.

### 2.6. Rapid Capsule Collection System (RaCCS)

The RaCCS is system that we designed to collect capsules while they are being produced. The inlet and outlet tubing of the RaCCS connect to the capsule collecting dish in the Spraybase electrosprayer, which is housed in a lucite box in a biosafety cabinet to maintain sterility. After the pump is turned on, capsules are collected onto a 100 µm filter (https://www.fishersci.com/shop/products/falcon-cell-strainers-4/0877119, accessed on 30 November 2022) washed by resuspension with HBSS, suspended in medium, transferred to a flask, and placed into a CO_2_ incubator at 37 °C.

### 2.7. Statistical Analysis

Data are represented as mean ± standard error of mean (SEM). One-way ANOVA followed by Tukey’s test or student *t*-test was used to analyze the data. A *p*-value < 0.05 is considered statistically significant.

## 3. Results

### 3.1. Effects of Encapsulated MSC In Vivo

#### 3.1.1. Encapsulated MSC in SCI

**Figure 1:**
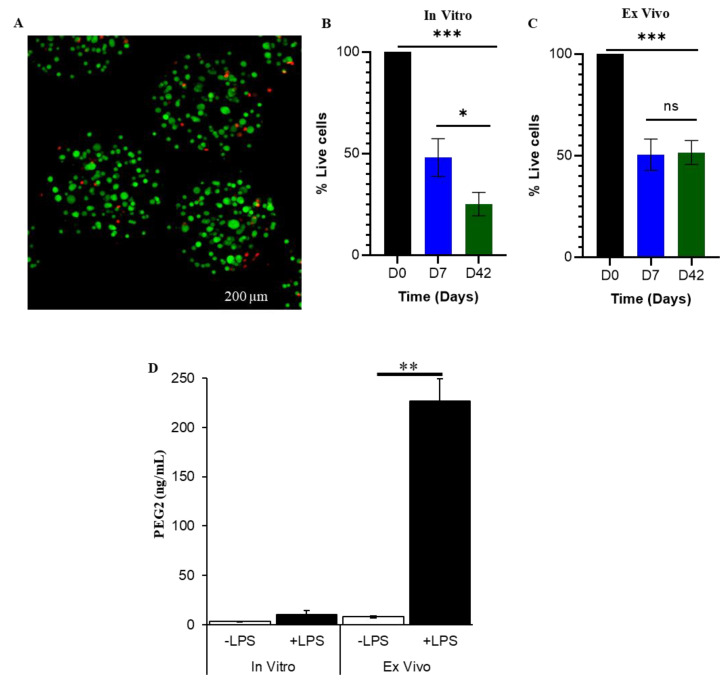
eMSC viability and biological activity in vitro and ex vivo. (**A**) In vitro eMSC live (green)/dead (red) staining same day (day 0) after preparation of eMSC; (**B**) Survival of eMSC in vitro (**C**) Survival of eMSC ex vivo after recovery from cauda equina of SCI rats; (cell number at day 0 = 86.0 ± 9.79, *n* = 6). On day 0, cells in eMSC were stained (number of cells/capsule = 56.6 ± 7.44, *n* = 5) after washing them from the needle hub post-injection. eMSC were incubated in vitro for 0, 7, and 42 days as noted, and eMSC from the same batches were injected in SCI rats and retrieved 7 and 42 days later (ex vivo), (**B**). The % of live cells was determined from confocal micrographs. One-way ANOVA followed by Tukey’s test was used to analyze the data (mean ± SEM). * *p* < 0.05 and *** *p* < 0.001. (**D**) PGE2 expression from eMSC in vitro and ex vivo after recovery from a 42-day incubation in vivo in the cauda equina as in (**C**). The eMSC recovered at 42 days were incubated for 24 h without LPS (−LPS) and after removing the conditioned medium it was replaced with fresh medium containing 1 µg/mL LPS (+LPS) for an additional 24 h. Student *t*-test was used for statistical analysis. ** *p* < 0.01.

At seven and 42 days after intrathecal injection of eMSC into the cauda equina [18] in injured rats, laminectomies were performed to expose the cauda equina and spinal cord so that free capsules could be recovered from these regions by washing with PBS. The recovered capsules were incubated in growth media and the number of live cells per capsule were counted (Figure 1A,B). Approximately the same number of live eMSC were found ex vivo per capsule when recovered 7 and 42 days after SCI (Figure 1B, ex vivo). Capsules from the same batch incubated in parallel but only in vitro had significantly lower numbers of live MSC per capsule than after 42 days in vivo (Figure 1B, in vitro vs. Figure 1C, ex vivo). This contrasts with the relatively constant numbers of cells per capsule maintained in vivo over the same period, indicating prolonged and perhaps superior survival of MSC over time in vivo compared to incubation in vitro. These results demonstrate that eMSC survive in the cauda equina of SCI rats for at least 42 days.

To analyze the secretory activity of eMSC after 42 days in the spinal canal, we recovered eMSC from the cauda equina and measured the level of PGE2, a critical anti-inflammatory prostaglandin, in the culture supernatant [11]. eMSC maintained only in vitro showed a 3.4-fold increase in PGE2 in response to LPS (Figure 1D). In an initial 24 h incubation period ex vivo in media without LPS (which activates MSC), the level of PGE2 secretion from 5000 MSC was relatively low falling between the levels detected for the same batch of eMSC treated with and without LPS maintained only in vitro (Figure 1D). Remarkably, very high levels of PGE2 (>20-fold) were detected ex vivo after a second 24 h incubation of the same eMSC in fresh medium containing LPS to activate the MSC (Figure 1D). The ability of LPS to activate high PGE2 secretion levels from eMSC ex vivo, suggests that eMSC retain responsiveness to changing inflammatory signals for at least 42 days in vivo and show highly enhanced expression upon restimulation.

**Figure 2:**
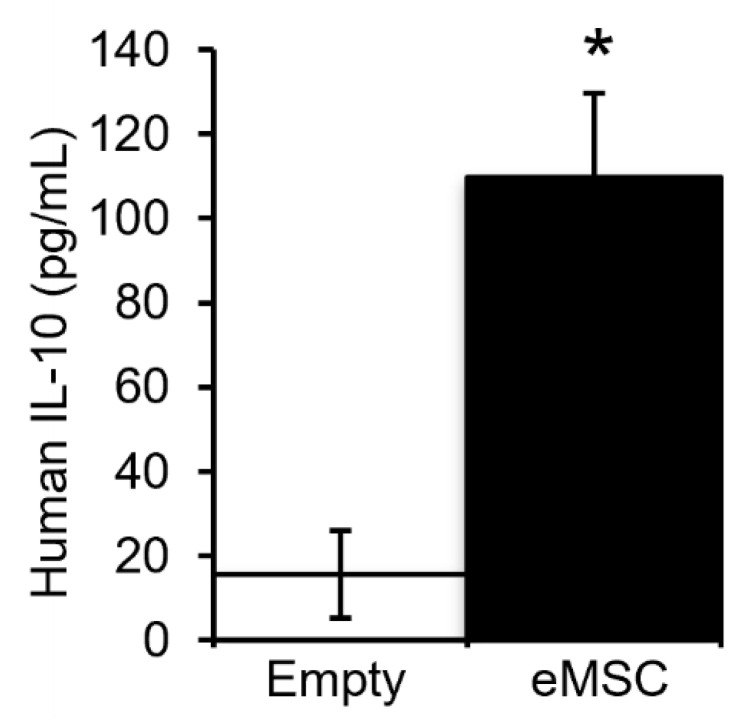
Measurement of human IL-10 in CSF 7 days after injection of eMSC into the cauda equina of SCI rats. Levels of human IL-10 were measured by ELISA in CSF collected from the foramen magnum at the base of the brain of SCI rats injected with capsules, without (empty) or with eMSC (*n* = 2/group). Student t-test was used for statistical analysis. * *p* < 0.05.

To determine whether cytokines released by human eMSC circulate rostrally towards the brain, CSF was collected from the foramen magnum at the base of the skull seven days after injection of human eMSC in the cauda equina. CSF produced in the choroid plexus flows caudally but at least some CSF recirculates rostrally back towards the brain driven by pulsatile blood flow and respiration [30]. ELISA for human IL-10 protein in rostral CSF detected ~100 pg/mL, which is significantly higher than the background level detected with empty capsules (<20 pg/mL) (Figure 2). These results suggest that intrathecal injection of human eMSC in the cauda equina below the caudal tip of the rat spinal cord resulted in human MSC-derived IL-10 in the most anterior region of the spinal cord. The rostral circulation of human IL-10 also suggests its presence in the CSF around the lumbar SCI site (It is not feasible to collect enough CSF from the rat injury site for analysis). The inferred presence of IL-10 in the SCI site likely contributes to the increase in CD206+ anti-inflammatory M2 macrophages and microglia after eMSC injection as we reported previously [18].

Considering that anti-inflammatory M2 macrophages secrete elevated levels of IL-10 compared to M1 macrophages [31], we measured levels of rat IL-10 mRNA in tissue dissected from rat SCI sites. qRT-PCR indicated elevated levels of rat IL-10 mRNA in the SCI site at seven days after injection of eMSC by comparison to control (data not shown).

#### 3.1.2. eMSC in Rat Endotoxemia

**Figure 3:**
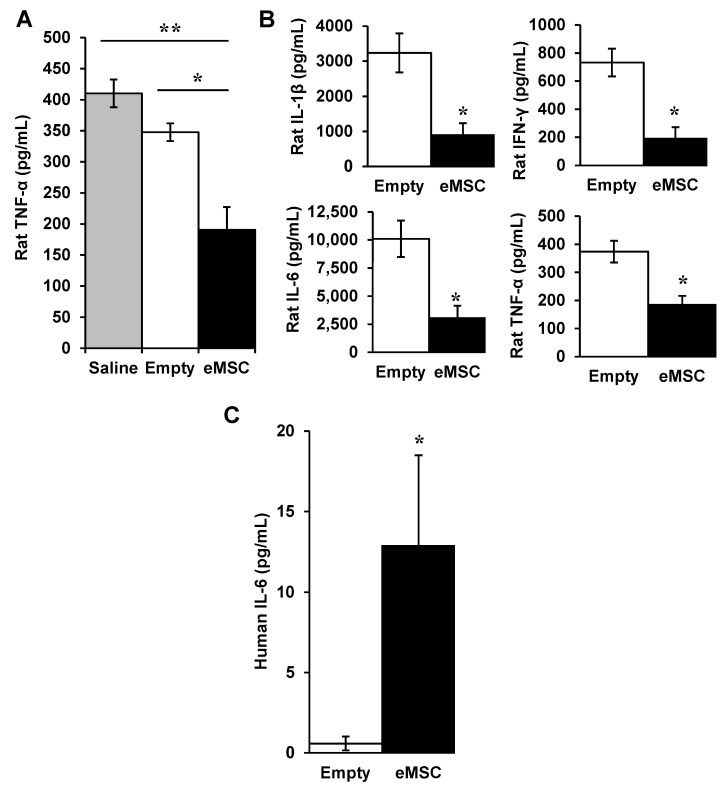
Effect of human eMSC in rat endotoxemia. (**A**) Expression of rat TNF-α in sera of rats injected with LPS and measured by ELISA after injection of saline alone or capsules without (empty) or with eMSC (*n* = 3/group). (**B**) Multiplex assay of sera from cardiac puncture showed significant reductions in rat IL-1β, IL-6, IFN-γ, and TNF-α. (**C**) Human IL-6 levels in the rat were detected at significantly higher levels than empty capsules control. *n* = 5 for Empty and *n* = 3 for eMSC. One-Way ANOVA or student *t*-test was done to test significance between the groups. * *p* < 0.05 and ** *p* < 0.01.

LPS-induced endotoxemia is another model for certain aspects of sepsis including cytokine storm, therefore we tested effects of eMSC in this animal model [10]. Rats were injected IP with LPS. Then, one hour later, they were injected IP with either saline or capsules with (eMSC) or without (empty). ELISA of the serum showed that treatment with eMSC reduced blood levels of TNF-α significantly (Figure 3A). Multiplex analysis for cytokines indicated that eMSC treatment reduced levels of several proinflammatory rat cytokines significantly including IL-1β, IFN-γ, IL-6, and TNF-α (Figure 3B). ELISA assays of serum also detected significantly elevated levels of human IL-6 (Figure 3C). Thus, human eMSC in the rat peritoneum released a human cytokine that was detected at elevated levels in sera within 5 h, demonstrating rapid systemic distribution using this method of eMSC delivery.

#### 3.1.3. Viability of MSC in Unpolymerized Alginate

There has been controversy whether extended exposure to high concentrations of unpolymerized alginate have deleterious effects on cells. However, we have not observed any reduction in MSC viability with incubation of MSC in 2.25% alginate for as long as 2 h (Figure S1). Thus, unpolymerized alginate is not a concern in our experiments.

### 3.2. A Rapid Capsule Collection System (RaCCS) for Scaling-Up Cell Encapsulation

#### 3.2.1. A Rapid Capsules Collection System (RaCCS)

**Figure 4:**
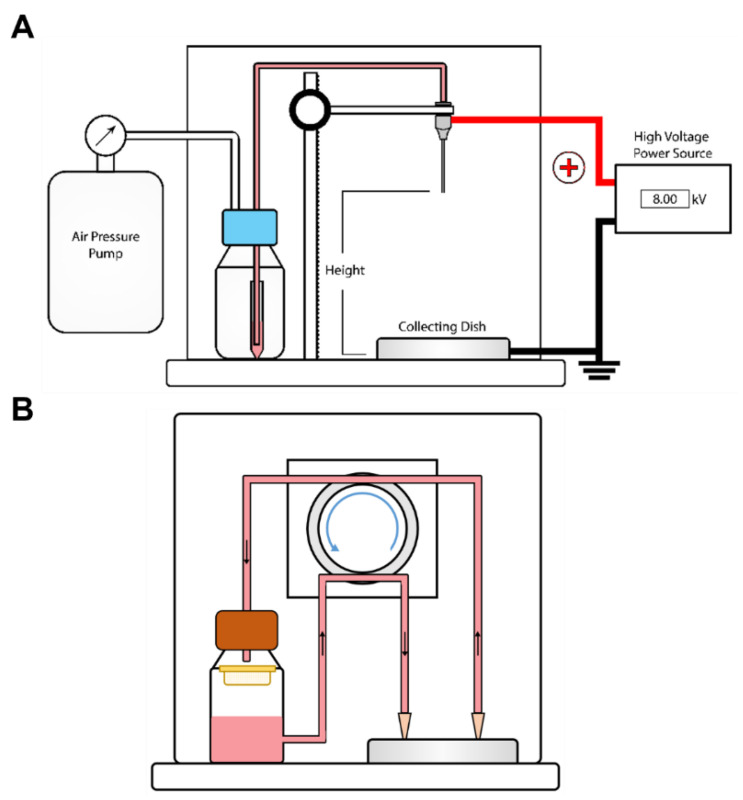
Schematic of Encapsulator and RaCCS. (**A**) An air pump is used with constant pressure to pressurize the vessel containing the alginate cell suspension, which provides force to drive it towards the needle. Spherical droplets fall in the electric field into the collecting dish where they are crosslinked. (**B**) The RaCCS consists of a peristaltic pump that drives crosslinking buffer into the dish through an inlet tube and the capsules are collected through an outlet tube onto a filter (yellow) and washed.

To scale-up, we have designed a flow system that enables collection of capsules from the crosslinking bath at any time during an encapsulation run. The RaCCS consists of an inlet port to flow crosslinking solution into the collection dish and an outlet port through which capsules are pumped onto a collecting filter for washing and post-encapsulation treatments (Figure 4). This enables rapid washing to terminate the crosslinking reaction. The flows are controlled by a peristaltic pump that can be turned on and off at any time during a run. Capsules can be collected within 15 s while the encapsulation run proceeds without interruption. This system provides tight control of crosslinking times in long runs by collecting and washing batches of capsules at intervals as the run proceeds.

#### 3.2.2. Properties of Encapsulation

**Figure 5:**
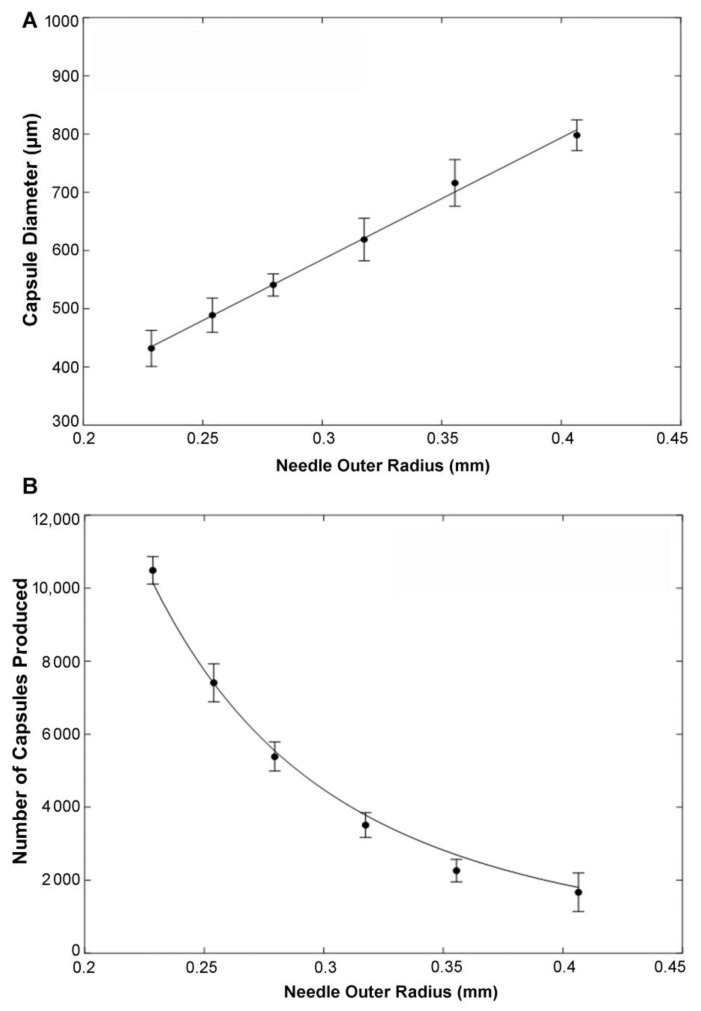
Capsule properties using constant force with a syringe pump. (**A**) Different needles with varying outer radii were used and diameters of the resulting capsules were measured. Capsule diameter is a linear function of needle outer radius; f(x) = 2092x − 43, R-squared = 0.9965; (**B**) Number of capsules produced is modeled as a function of needle outer radius, f(x) = (0.2021x) − 3, R-squared = 0.9918.

Encapsulation parameters have been reported recently using a piston-driven constant force loading mode [32]. Using the same mode, we compared parameters optimized for encapsulation of chondrocytes with those for that we obtained for MSC. Gansau et al., determined that by comparison to 1 and 3% alginate, 2% (*w*/*v*) alginate was optimal to yield uniformly shaped spherical capsules. This is close to the 2.25% that we have used to maximize capsule stability and permeability to cytokines from MSC [18,32] and we used this concentration in all our current experiments. Input cell densities in the unpolymerized alginate in both cases yielded maximal cell incorporation into the capsules at inputs of ~5 million cells/mL, with higher cell input concentrations yielding lower percent cell incorporation [32]. We also used a flow rate of 5 mL/h [18] because mis-shapened capsules were found at higher flow rates [32]. Using these parameters, capsule diameters were reported to increase with needle diameters [32] and we found a linear relationship using six different needles with capsule diameters increasing from 432 ± 31 µm to 798 ± 26 µm, for 26 G to 21 G needles (needle gauge (G) is inversely related to needle diameter), respectively (Figure 5A). The numbers of capsules (3-dimensional spheres) generated decreased with increasing needle radii in an exponential manner as expected because capsule volume is related to the cube of the capsule radius (r^3^) (Figure 5B).

**Figure 6:**
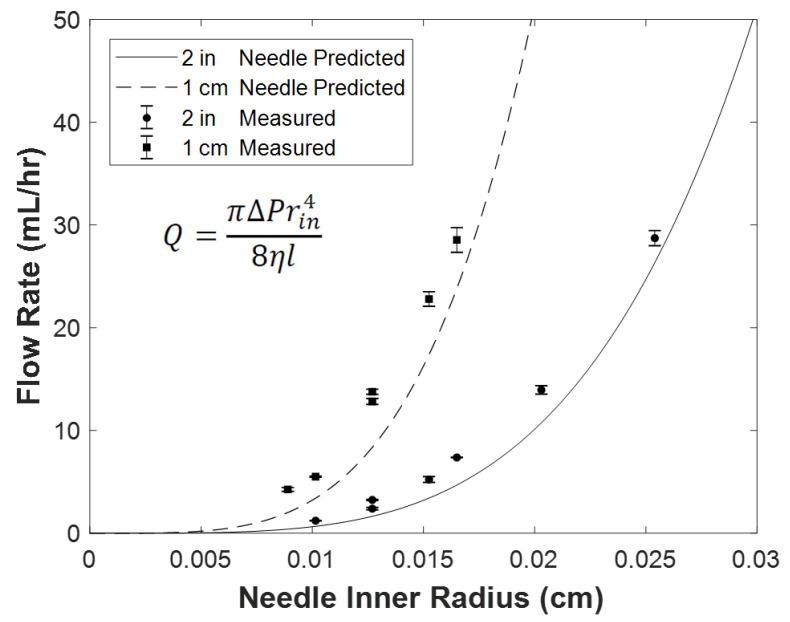
Flow rates increase exponentially as a function of needle inner radius. The viscosity of 2.25% (*w*/*v*) alginate was measured to be 330 mPas and used to calculate the predicted curves. The flow rate is proportional to the 4th power of needle inner radius in agreement with the Hagen Poiseuille Equation (Appendix A).

Next, we compared the constant flowrate to constant pressure modes of the encapsulator to deliver cells in unpolymerized alginate to the injection needles. In the constant flowrate mode, pressure increases as the run progresses particularly in long runs to reach levels high enough that connections between the tubing can fail. This is not a problem with the constant pressure mode as the pressure is fixed. The flow rate increases exponentially with increasing inner radii of the needle (Figure 6). Therefore, relatively high flow rates can be achieved in the constant pressure mode. The relationship between the inner needle radius and the flow rate can be modeled using Hagen-Poiseuille Equation with good correlation for our data using 1- and 2-inch-long needles. The flow rates are lower for the 2- vs. the 1-inch needles because the longer length of the needle contributes more resistance to flow. These properties of the constant pressure mode provide a more dynamic range of configurations than the constant flow mode for rapid encapsulation and scale-up.

For development of the RaCCS, we used GFP + C6 cells [26] because their growth is rapid, they are inexpensive, and they express GFP, which allows fluorescence visualization without any additional treatment to measure numbers of live cells/capsule. After we optimized the parameters as described above, human MSC were encapsulated using the RaCCS and the incorporation of live cells into the capsules was measured. The preparation size was held constant at 1 mL and the cell input ranged from 5–7.5 × 10^6^. The percent of MSC incorporated in 3 experiments was 60% resulting in eMSC with 230 cells/capsule in capsules of 440 µm in diameter. The percent of cells incorporated decreases above ~5 × 10^6^ cells/mL. To evaluate scale-up using the RaCCS, we encapsulated 36 million CHO cells in an alginate suspension of 5 mL in a single 60 min run and achieved a yield of >90% incorporation. This level of sale up demonstrates feasibility for preparing doses to treat humans in clinical trials.

## 4. Discussion

The registration at ClinicalTrials.gov of greater than one thousand and five hundred clinical trials testing MSC for various indications [33], most recently including COVID-19 [34], underscores the tremendous interest in therapeutics with these cells. The use of eMSC by lumbar puncture generated changes in immunomodulatory factors such as PGE2 suggesting that secreted factors suppress cytokine storm acutely in vivo and promote improved outcomes. Several factors have prevented eMSC technology from moving to the clinic including the failure of long-term eMSC survival due to foreign body reactions, for which there are solutions [14,35]. There are also limitations in scale-up to produce sufficient quantities of eMSC for treating patients and we describe herein a solution to the problem of scale-up using a novel design for encapsulation and recovery of capsules.

There appears to be a loss of approximately half of eMSC within the first week in vivo and this survival level was maintained in vivo for at least 6 weeks. The surviving MSC after 6 weeks secreted a slightly higher level of the anti-inflammatory prostaglandin PGE2 [11] ex vivo than eMSC maintained only in vitro without LPS activation, indicating the cells maintained secretory activity in vivo. However, when activated with LPS ex vivo, the eMSC expressed highly elevated levels of PGE2, suggesting that exposure to the inflammatory environment in vivo primed them for subsequent responsiveness, demonstrating that eMSC is a bio-responsive system. This dramatic effect should be considered as preliminary insofar as very limited numbers of capsules were retrieved from two rats and then pooled for a single experiment. Additional studies are needed to determine the extent and timing of eMSC responsiveness in vivo. Similar experiments are not feasible with free MSC as they cannot be recovered from the body after injection [36].

The action of the eMSC must be via secreted factors given that the cells are retained in the capsules for at least for 6 weeks. Considering that the capsules are injected below the end of the spinal cord, the improved recovery in the injury site and locomotion [19] indicate that eMSC act at a distance. The increased expression of rat IL-10 mRNA in the injury site is likely to result from injection of human eMSC at a distance from the SCI site in the cauda equina. This is in contrast to free MSC, which migrate extensively but survive for only a few days after injection [36]. We have also observed that intraperitoneal injection of eMSC in rodent models of sepsis reduced serum levels of TNFα, confirming that eMSC act a distance (unpublished observations). Thus, eMSC are a better designed system than free MSC for long-term survival, making long-term treatment in chronic diseases involving inflammation feasible.

The systemic effects demonstrated by changes in cytokines in the sera after IP eMSC injection is another example of eMSC action at a distance. The effect of eMSC by IP to generate changes in cytokines in blood is novel. Among the many clinical trials testing MSC, multiple doses are often provided two days to one week after an initial dose [33]. It is likely that eMSC will be advantageous because the cells survive longer than with free MSC and may not need additional dosing for several weeks. MSC have been demonstrated to save lives in graft vs. host disease [37], ARDS [38], and COVID-19 [34], and eMSC may be more effective as a bio-responsive therapy.

MSC are produced by many organizations and companies in very large quantities to inject hundreds of millions of cells per patient in clinical trials [33]. eMSC are effective in several animal models of disease and injury, but this technology has not been translated to the clinic so far in part because a method for scale-up has not been devised. We have invented a RaCCS that enables scale-up to yield 36 million cells in a preliminary experiment. Considering that 30,000 eMSC produced a similar response to 250,000 free MSC in rat SCI [19], encapsulation of 36 million eMSC should be equivalent to ~300 million eMSC, which is enough to treat at least two patients with a minimal effective dose of 150 million free MSC [33]. In any case, these estimates indicate that eMSC can be produced in sufficient doses for use in at least small clinical trials.

It has been suggested that MSC apoptosis and efferocytosis plays a role in the anti-inflammatory action of MSC in graft vs. host disease [37]. This should occur in less than one week after injection since MSC are barely detectable thereafter [36]. It is possible that loss of ~1/3 of eMSC that we observed within the first week in the injured spinal cord may be due to apoptosis. However, dead cells were rarely observed by Live/Dead assay in capsules retrieved from the spinal cord at one or six weeks after delivery. Thus, long-term effects of eMSC are likely attributable to secreted factors.

The ability of eMSC to reduce blood levels of pro-inflammatory cytokines within 5 h in the LPS-induced endotoxicity underscores the rapidity of the response to eMSC injected intraperitoneally, outside the bloodstream in the rat. However, this is not the best model for sepsis. In preliminary studies we found using a more appropriate model for sepsis, i.e., mouse cecal ligation and puncture, that human eMSC reduced levels of IL-6 and TNF-α in sera after 16 h treatments with eMSC (unpublished observation MG, SB, MK). The combined results suggest than eMSC may be useful for acute treatment of cytokine storms that occurs in many inflammatory disorders including COVID-19 [34].

The constant pressure mode of the encapsulator is advantageous over the constant flowrate mode because tubing connections can fail as the pressure rises especially in long runs to scale up production of capsules. When using constant pressure, one should first determine a high pressure that does not compromise cell viability and then determine needle length, needle outer-diameter, and needle inner-diameter as desired. As inner needle diameters increase, flow rates increase to the power of 4. Shortening the needle increases the flow rate linearly. Shorter and wider diameter needles have higher flow rates, yielding larger capsules with high cell yields in shorter run times. Optimizing these parameters may increase yields in encapsulations.

Capsules with diameters larger than 0.5 mm are difficult to inject through syringes unless their caliber is very large because they tend to aggregate. Two ways to keep the capsule size relatively low is to decrease the needle outer diameter and increase the applied voltage. Beveled needles have smaller outer diameters than blunt ones, thus producing smaller capsules without lowering the flow rates [19]. High electric fields do not decrease the viability of cells in microcapsules due to the Faraday cage effect and it has been reported that voltages as high as 30 kV do not decrease viability [39,40]. The downside of using high voltages is increased needle vibration as we observed with the 2-inch 27 G needle at 8.0 kV. This effect can be minimized using shorter needle lengths.

The most widely used electrostatic cell encapsulator is produced by Nisco, Zurich, Switzerland (http://www.nisco.ch/var_v1.htm, accessed on 30 November 2022). In this system, cells suspended in alginate monomers are driven by a syringe pump at a constant flow rate to extrude droplets from a needle. The unpolymerized alginate droplets are driven under an electrostatic potential into a collecting vessel where they are crosslinked by 20–100 mM divalent cations in a solution that is mixed using a stir bar to prevent capsules from clumping. At the conclusion of the run, the apparatus is disassembled to retrieve the capsules from the collection bath for further processing including post-encapsulation treatments and washing into cell culture media. Other encapsulators from Buchi, Flawil, Switzerland (https://www.buchi.com/us-en/products/spray-drying-and-encapsulation/encapsulator-b-395-pro, accessed on 30 November 2022) and Inotech, Flawil, Switzerland (http://www.encap.ch/, accessed on 30 November 2022) have more complex designs for batch stirring and collection but they also are closed systems that do not allow sampling or capsule collection until the run is terminated.

Given that relatively high concentrations of divalent cations used for crosslinking and unpolymerized alginate may be detrimental to cells, it is important to transfer capsules into physiological buffers as soon as possible after encapsulation. One also needs to consider that the duration of the encapsulation run can result in large differences in crosslinking times and exposure to divalent cations between formation of the first and the last capsule that are unavoidable with batch reactors. For this reason, protocols using batch reactors often add a post-crosslinking period to ensure sufficient cross-linking for capsule stability while reducing relative differences in total crosslinking times among the capsules. Nevertheless, capsules will be subject to different exposure times that introduce heterogeneity in the population. Although encapsulation details are often not reported, the shortest run times and hence minimal crosslinking times appear to range between 20–30 min using up to ~1 mL of alginate with ~1–20 million cells [16]. This yields several millions of encapsulated cells, which are sufficient for studies with rodents but not enough for large mammals. Scale-up is problematic with these closed system encapsulators because they require longer run times that will increase heterogeneity further.

## 5. Conclusions

In summary, eMSC may be advantageous for treating SCI because additional dosing may not be required for weeks, and lower doses are effective by comparison to free MSC. Delivery of eMSC to the CNS by lumbar puncture is effective in SCI and may be applicable to other neurological disorders because cytokines appear to circulate in the CNS into the foramen magnum. Efficacy of IP eMSC delivery in sepsis may provide a convenient method to supply eMSC secreted factors systemically as they generate rapid changes in cytokines in the blood. Finally, the feasibility of scaling eMSC production may provide novel therapies requiring fewer MSC with longer-lasting activity in various inflammatory diseases.

## Data Availability

The data presented in this study are available on request from the corresponding author.

## Supplementary Materials

The following supporting information can be downloaded at: https://www.mdpi.com/article/10.3390/biom12121803/s1, Figure S1: Survival of MSC in Alginate over Time. MSC were plated at a density of 5000 cells per 96 well in DMEM without Calcium, which cannot be used because it crosslinks alginate. The cells were washed with DMEM without Calcium and 2.25% alginate were added per well in DMEM without Calcium. After the times indicated, the alginate medium was removed, and the cells were washed with PBS 3 times. Medium was replaced with stain for Calcein and Ethidium (ThermoFisher) in α-MEM Media plus 10% FBS. After 30 min, cells were washed and imaged by confocal microscopy. Assays were performed in triplicate wells, 500 cells per well per condition and averages were plotted. ANOVA showed no significant difference between groups.

## Appendix A

Alginate Microencapsulation using Spraybase: A 2.25% (*w*/*v*) alginate solution was loaded into a 10 mL syringe (BD Biosciences) and attached to a syringe pump (Harvard Apparatus 22). A 1 mm inner diameter PTFE tubing (Spraybase) was attached to the syringe which was then connected to a needle. Alginate beads were generated using an electrostatic bead generator (Spraybase) at a flow rate of 5 mL/h, an applied voltage of 8.0 kV, using a needle height of 5 cm from the bottom of the dish at room temperature. The extruded droplets of alginate were driven electrostatically into a 50 mL collecting vessel containing 50 mM BaCl2 (Sigma-Aldrich) 145 mM NaCl, 10 mM MOPS, and 14 mM Glucose where they were polymerized for 10 min at room temperature. Microcapsules were washed with HEPES Buffer, allowed to settle under gravity, re-suspended in PBS and incubated in alpha-MEM complete media.

Alginate Microencapsulation: The 2.25% (*w*/*v*) alginate solution (with or without cells) was transferred to a 15 mL tube (Falcon Corning) and placed into a 500 mL pressure chamber (Duran 250 mL). A 1 mm diameter PTFE tubing was placed at the bottom of the alginate solution which was then connected to a needle. At room temperature alginate droplets were generated using a Spraybase electrostatic encapsulator at a constant positive gauge pressure of 0.75 bar, an applied voltage of 8.0 kV, and a needle height of 5 cm. The extruded droplets of alginate were driven electrostatically into a 50 mL collecting vessel containing 50 mM BaCl2, 145 mM NaCl, 10 mM MOPS, and 14 mM Glucose where they were polymerized for 10 min at room temperature. Spherical microcapsules were washed with HEPES Buffer, allowed to settle under gravity, and were re-suspended in PBS Buffer.

Flow Rates using Needles with Different Gauges at Constant Pressure: Pressure controlled alginate micro-encapsulations with different configurations of needles were performed. A constant volume of 2.25% (*w*/*v*) of sodium alginate was pumped through a select needle using the protocol above. Time was started at the formation of the first droplet and stopped at the last. This time interval and the volume were used to calculate the average flow rate of the system which was repeated 3 times per needle.

Diameter and Number of Capsules using Different Needle Gauges at Constant Flowrates: Alginate microencapsulation at constant flowrate with different gauge needles was performed to determine the relationship between the outer needle and capsule diameters. Aliquots of capsules were imaged in an inverted microscope (IX81, Olympus, Tokyo, Japan) at 10× magnification and diameters for 100 capsules were measured per condition using the Axiovision Program. The number of capsules were also counted to determine the total number of capsules produced.

Understanding the relationship between flow rate, pressure, and needle inner radius is necessary to pick the ideal parameters for cell encapsulation. The Hagen-Poiseuille Equation can predict this correlation:

Q = (πΔPr_in_^4^)/8ηl

where Q is the flow rate, ΔP is the gauge pressure, r_in_ is the inner radius of needle, η is the viscosity of alginate, and l is the length of needle.

Alginate capsules are usually limited to ~1 mm because it has been suggested that larger capsules limit perfusion of nutrients [41], which can cause cells in the center of the capsule to die. Predicting the size of capsules produced is useful for particular applications. The major forces acting on the alginate droplet at the tip of the needle are: surface tension, gravity, and electric force. The magnitude of the normal electric field at the tip of the stainless steel needle can be approximated by [42,43,44]:

E_n_ = (√2 V)/(r_out_ ln(4H/r_out_)

where r_out_ is the outer radius of the needle, V is the applied voltage, H is the distance between the nozzle tip and grounded collecting dish. The force on the droplet in a non-uniform electric field is [43,44]:

F_E_ = 1/2 S∙ε_n_^2^ = 4πε_0_V^2^/ln^2^(4H/r_out_)

where S is the surface area of the droplet, approximated by 2πr_out_^2^. Predicting the size of a capsule can be done with a modified Tate’s Law. After including the force of the electric field and the droplet shrinking factor due to crosslinking, the overall mass balance for a droplet formation in a non-uniform electric field can be approximated by [44]:

4πε_0_V^2^/ln^2^(4H/r_out_) + ρg πd^3^/6 = 2π γφ r_out_/K_SF_

d = K_SF_∙∛(12/ρg (r_out_γφ − 2ε_0_ (V^2^/ln^2^(4H/r_out_))^2^))

where d is the capsule diameter, γ is the surface tension of alginate in air, ρ is density of alginate, K_SF_ is the shrinking factor, and φ is the Harkins correction factor. As shown in the above equation, capsule size decreases with decreasing needle outer diameter, higher voltage, and lower height.

## References

[1] Matthay M.A., Pati S., Lee J.-W. (2017). Concise Review: Mesenchymal Stem (Stromal) Cells: Biology and Preclinical Evidence for Therapeutic Potential for Organ Dysfunction Following Trauma or Sepsis. Stem Cells.

[2] Prockop D.J., Kota D.J., Bazhanov N., Reger R.L. (2010). Evolving Paradigms for Repair of Tissues by Adult Stem/Progenitor Cells (MSCs). J. Cell Mol. Med..

[3] Prockop D.J. (2017). The Exciting Prospects of New Therapies with Mesenchymal Stromal Cells. Cytotherapy.

[4] de Almeida D.C., Donizetti-Oliveira C., Barbosa-Costa P., Origassa C.S., Câmara N.O. (2013). In Search of Mechanisms Associated with Mesenchymal Stem Cell-Based Therapies for Acute Kidney Injury. Clin. Biochem. Rev..

[5] Maron-Gutierrez T., Laffey J.G., Pelosi P., Rocco P.R.M. (2014). Cell-Based Therapies for the Acute Respiratory Distress Syndrome. Curr. Opin. Crit. Care.

[6] Alijotas-Reig J., Esteve-Valverde E., Belizna C., Selva-O’Callaghan A., Pardos-Gea J., Quintana A., Mekinian A., Anunciacion-Llunell A., Miró-Mur F. (2020). Immunomodulatory Therapy for the Management of Severe COVID-19. Beyond the Anti-Viral Therapy: A Comprehensive Review. Autoimmun. Rev..

[7] Cavaillon J.-M. (2018). Exotoxins and Endotoxins: Inducers of Inflammatory Cytokines. Toxicon.

[8] Huet O., Chin-Dusting J.P.F. (2014). Septic Shock: Desperately Seeking Treatment. Clin. Sci..

[9] Sakr Y., Jaschinski U., Wittebole X., Szakmany T., Lipman J., Ñamendys-Silva S.A., Martin-Loeches I., Leone M., Lupu M.-N., Vincent J.-L. (2018). Sepsis in Intensive Care Unit Patients: Worldwide Data from the Intensive Care over Nations Audit. Open Forum Infect. Dis..

[10] Lombardo E., van der Poll T., DelaRosa O., Dalemans W. (2015). Mesenchymal Stem Cells as a Therapeutic Tool to Treat Sepsis. World J. Stem Cells.

[11] Prockop D.J., Oh J.Y. (2012). Mesenchymal Stem/Stromal Cells (MSCs): Role as Guardians of Inflammation. Mol. Ther..

[12] Qi M., Strand B.L., Mørch Y., Lacík I., Wang Y., Salehi P., Barbaro B., Gangemi A., Kuechle J., Romagnoli T. (2008). Encapsulation of Human Islets in Novel Inhomogeneous Alginate-Ca^2+^/Ba^2+^ Microbeads: In Vitro and in Vivo Function. Artif. Cells Blood Substit. Immobil. Biotechnol..

[13] Paredes Juárez G.A., Spasojevic M., Faas M.M., de Vos P. (2014). Immunological and Technical Considerations in Application of Alginate-Based Microencapsulation Systems. Front. Bioeng. Biotechnol..

[14] Vegas A.J., Veiseh O., Doloff J.C., Ma M., Tam H.H., Bratlie K., Li J., Bader A.R., Langan E., Olejnik K. (2016). Combinatorial Hydrogel Library Enables Identification of Materials That Mitigate the Foreign Body Response in Primates. Nat. Biotechnol..

[15] Gurruchaga H., Saenz del Burgo L., Ciriza J., Orive G., Hernández R.M., Pedraz J.L. (2015). Advances in Cell Encapsulation Technology and Its Application in Drug Delivery. Expert Opin Drug Deliv..

[16] Levit R.D., Landázuri N., Phelps E.A., Brown M.E., García A.J., Davis M.E., Joseph G., Long R., Safley S.A., Suever J.D. (2013). Cellular Encapsulation Enhances Cardiac Repair. J. Am. Heart Assoc..

[17] Landázuri N., Levit R.D., Joseph G., Ortega-Legaspi J.M., Flores C.A., Weiss D., Sambanis A., Weber C.J., Safley S.A., Taylor W.R. (2016). Alginate Microencapsulation of Human Mesenchymal Stem Cells as a Strategy to Enhance Paracrine-Mediated Vascular Recovery after Hindlimb Ischaemia. J. Tissue Eng. Regen. Med..

[18] Barminko J., Kim J.H., Otsuka S., Gray A., Schloss R., Grumet M., Yarmush M.L. (2011). Encapsulated Mesenchymal Stromal Cells for in Vivo Transplantation. Biotechnol. Bioeng..

[19] Kumar S., Babiarz J., Basak S., Kim J.H., Barminko J., Gray A., Mendapara P., Schloss R., Yarmush M.L., Grumet M. (2015). Sizes and Sufficient Quantities of MSC Microspheres for Intrathecal Injection to Modulate Inflammation in Spinal Cord Injury. Nano Life.

[20] Tobias C.A., Han S.S.W., Shumsky J.S., Kim D., Tumolo M., Dhoot N.O., Wheatley M.A., Fischer I., Tessler A., Murray M. (2005). Alginate Encapsulated BDNF-Producing Fibroblast Grafts Permit Recovery of Function after Spinal Cord Injury in the Absence of Immune Suppression. J. Neurotrauma.

[21] Grandoso L., Ponce S., Manuel I., Arrúe A., Ruiz-Ortega J.A., Ulibarri I., Orive G., Hernández R.M., Rodríguez A., Rodríguez-Puertas R. (2007). Long-Term Survival of Encapsulated GDNF Secreting Cells Implanted within the Striatum of Parkinsonized Rats. Int. J. Pharm..

[22] Goren A., Dahan N., Goren E., Baruch L., Machluf M. (2010). Encapsulated Human Mesenchymal Stem Cells: A Unique Hypoimmunogenic Platform for Long-Term Cellular Therapy. FASEB J..

[23] Klinge P.M., Harmening K., Miller M.C., Heile A., Wallrapp C., Geigle P., Brinker T. (2011). Encapsulated Native and Glucagon-like Peptide-1 Transfected Human Mesenchymal Stem Cells in a Transgenic Mouse Model of Alzheimer’s Disease. Neurosci. Lett..

[24] Detante O., Moisan A., Dimastromatteo J., Richard M.-J., Riou L., Grillon E., Barbier E., Desruet M.-D., De Fraipont F., Segebarth C. (2009). Intravenous Administration of 99mTc-HMPAO-Labeled Human Mesenchymal Stem Cells after Stroke: In Vivo Imaging and Biodistribution. Cell Transpl..

[25] Mørch Y.A., Donati I., Strand B.L., Skjåk-Braek G. (2006). Effect of Ca^2+^, Ba^2+^, and Sr^2+^ on Alginate Microbeads. Biomacromolecules.

[26] Friedlander D.R., Brittis P.A., Sakurai T., Shif B., Wirchansky W., Fishell G., Grumet M. (1998). Generation of a Radial-like Glial Cell Line. J. Neurobiol..

[27] Moran D.M., Koniaris L.G., Jablonski E.M., Cahill P.A., Halberstadt C.R., McKillop I.H. (2006). Microencapsulation of Engineered Cells to Deliver Sustained High Circulating Levels of Interleukin-6 to Study Hepatocellular Carcinoma Progression. Cell Transplant.

[28] Hasegawa K., Chang Y.-W., Li H., Berlin Y., Ikeda O., Kane-Goldsmith N., Grumet M. (2005). Embryonic Radial Glia Bridge Spinal Cord Lesions and Promote Functional Recovery Following Spinal Cord Injury. Exp. Neurol..

[29] Yagi H., Soto-Gutierrez A., Parekkadan B., Kitagawa Y., Tompkins R.G., Kobayashi N., Yarmush M.L. (2010). Mesenchymal Stem Cells: Mechanisms of Immunomodulation and Homing. Cell Transpl..

[30] Brinker T., Stopa E., Morrison J., Klinge P. (2014). A New Look at Cerebrospinal Fluid Circulation. Fluids Barriers CNS.

[31] Ylöstalo J.H., Bartosh T.J., Coble K., Prockop D.J. (2012). Human Mesenchymal Stem/Stromal Cells Cultured as Spheroids Are Self-Activated to Produce Prostaglandin E2 That Directs Stimulated Macrophages into an Anti-Inflammatory Phenotype. Stem Cells.

[32] Gansau J., Kelly L., Buckley C.T. (2018). Influence of Key Processing Parameters and Seeding Density Effects of Microencapsulated Chondrocytes Fabricated Using Electrohydrodynamic Spraying. Biofabrication.

[33] Kabat M., Bobkov I., Kumar S., Grumet M. (2020). Trends in Mesenchymal Stem Cell Clinical Trials 2004-2018: Is Efficacy Optimal in a Narrow Dose Range?. Stem Cells Transl. Med..

[34] Grumet M., Sherman J., Dorf B.S. (2022). Efficacy of MSC in Severe COVID-19 Patients: A Review and a Case Study. Stem Cells Transl. Med.

[35] Bochenek M.A., Veiseh O., Vegas A.J., McGarrigle J.J., Qi M., Marchese E., Omami M., Doloff J.C., Mendoza-Elias J., Nourmohammadzadeh M. (2018). Alginate Encapsulation as Long-Term Immune Protection of Allogeneic Pancreatic Islet Cells Transplanted into the Omental Bursa of Macaques. Nat. Biomed. Eng..

[36] Wagner B., Henschler R. (2013). Fate of Intravenously Injected Mesenchymal Stem Cells and Significance for Clinical Application. Adv Biochem. Eng. Biotechnol..

[37] Cheung T.S., Bertolino G.M., Giacomini C., Bornhäuser M., Dazzi F., Galleu A. (2020). Mesenchymal Stromal Cells for Graft Versus Host Disease: Mechanism-Based Biomarkers. Front Immunol..

[38] Bellingan G., Jacono F., Bannard-Smith J., Brealey D., Meyer N., Thickett D., Young D., Bentley A., McVerry B.J., Wunderink R.G. (2022). Safety and Efficacy of Multipotent Adult Progenitor Cells in Acute Respiratory Distress Syndrome (MUST-ARDS): A Multicentre, Randomised, Double-Blind, Placebo-Controlled Phase 1/2 Trial. Intensive Care Med..

[39] Gryshkov O., Pogozhykh D., Zernetsch H., Hofmann N., Mueller T., Glasmacher B. (2014). Process Engineering of High Voltage Alginate Encapsulation of Mesenchymal Stem Cells. Mater. Sci. Eng. C Mater. Biol. Appl..

[40] Huang H., Sun M., Heisler-Taylor T., Kiourti A., Volakis J., Lafyatis G., He X. (2015). Stiffness-Independent Highly Efficient On-Chip Extraction of Cell-Laden Hydrogel Microcapsules from Oil Emulsion into Aqueous Solution by Dielectrophoresis. Small.

[41] Orive G., Santos E., Pedraz J.L., Hernández R.M. (2014). Application of Cell Encapsulation for Controlled Delivery of Biological Therapeutics. Adv. Drug Deliv. Rev..

[42] Nedović V.A., Obradović B., Leskošek-Čukalović I., Trifunović O., Pešić R., Bugarski B. (2001). Electrostatic Generation of Alginate Microbeads Loaded with Brewing Yeast. Process Biochem..

[43] Rahman K., Ko J.-B., Khan S., Kim D.-S., Choi K.-H. (2010). Simulation of Droplet Generation through Electrostatic Forces. J. Mech. Sci. Technol..

[44] Lee S.-H., Nguyen X.H., Ko H.S. (2012). Study on Droplet Formation with Surface Tension for Electrohydrodynamic Inkjet Nozzle. J. Mech. Sci. Technol..

