# Supplementary material for: Anti-Inflammatory Effects of Encapsulated Human Mesenchymal Stromal/Stem Cells and a Method to Scale-Up Cell Encapsulation
Source: Biomolecules. 2022 Dec 2;12(12):1803. doi: 10.3390/biom12121803 (PMC9775968; doi:10.3390/biom12121803)
Supplement: Supplementary file 1 [file biomolecules-12-01803-s001.zip › biomolecules-2060230-supplementary.pdf]

Figure S1

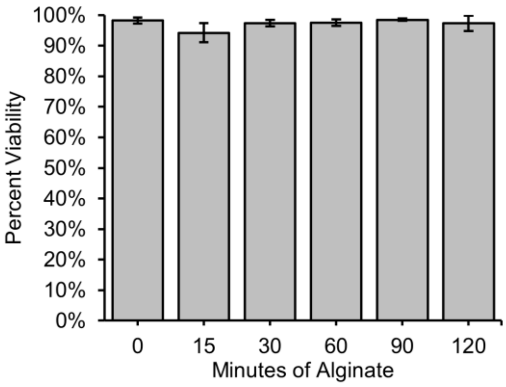

Figure S1: Survival of MSC in Alginate over Time. MSC were plated at a density of 5,000 cells per 96 well in DMEM without Calcium, which cannot be used because it crosslinks alginate. The cells were washed with DMEM without Calcium and 2.25% alginate were added per well in DMEM without Calcium. After the times indicated, the alginate medium was removed, and the cells were washed with 1x PBS 3 times. Medium was replaced with stain for Calcein and Ethidium (ThermoFisher) in α-MEM Media plus 10% FBS. After 30 minutes, cells were washed and imaged by confocal microscopy. In triplicate well, 500 cells per well per condition and averages were plotted. ANOVA showed no significant difference between groups.
